# P-1146. Environmental Surfaces in Healthy Households as Reservoirs of Extended-Spectrum Cephalosporin-Resistant Enterobacterales in the Community

**DOI:** 10.1093/ofid/ofae631.1333

**Published:** 2025-01-29

**Authors:** Catherine Babbs, Barrett Breeze, Mary G Boyle, Ahmed Babiker, Ainsley Heidbreder, Stefan J Green, Stephanie A Fritz, Latania K Logan

**Affiliations:** Emory School of Medicine, Atlanta, Georgia; Emory University School of Medicine, Roswell, Georgia; Washington University School of Medicine, St. Louis, Missouri; Emory University, Atlanta, GA; Washington University at St. Louis, St. Louis, Missouri; Rush University Medical Center, Chicago, Illinois; Washington University School of Medicine, St. Louis, Missouri; Emory University School of Medicine, Roswell, Georgia

## Abstract

**Background:**

Community-acquired extended-spectrum cephalosporin resistant (ESCr)-Enterobacterales (E) are increasing in healthy populations, yet the sources of acquisition are unknown. We assessed household (HH) environmental surface samples (HS) in St. Louis, MO, to identify the surfaces most frequently contaminated with ESCr-E and the associated mechanisms of antibiotic resistance (AR).

**Figure 1.**
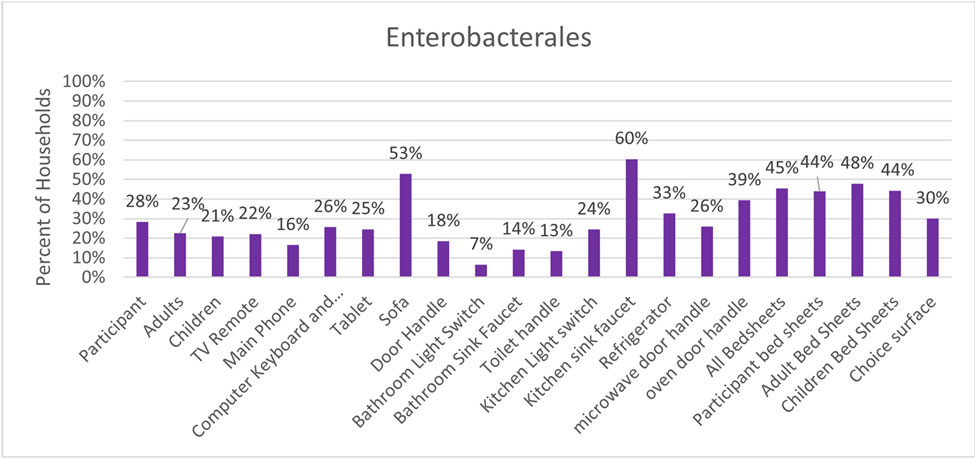
Enterobacterales within households.

**Methods:**

From 139 HHs, samples from 21 HS and inguinal fold (IF) samples of healthy adults and children were cultured for bacteria. Families were recruited from outpatient pediatric settings. Bacterial identification and antibiotic susceptibility testing were conducted (Vitek^®^2, bioMérieux). Molecular characterization of ESCr-E was performed by PCR detection of beta-lactamase (*bla*) and plasmid-mediated quinolone resistance (PMQR) genes. Whole genome sequencing (WGS) was performed on select HH with > 1 colonized HS to evaluate for genetic relatedness, plasmids, and AR genes. A HH cluster was defined as < 15 single nucleotide polymorphism differences between 2 isolates.

**Figure 2.**
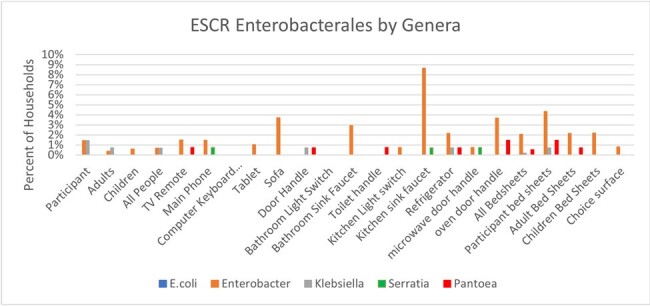
ESCR Enterobacterales by genera

**Results:**

Of 581 IF and 2801 HS, the majority harbored bacteria (IF, 97-100% and HS, 79-100%) and gram-negative bacteria, (IF, 72-80% and HS, 75-87%). IF colonization with Enterobacterales was ∼23% and ranged from 7-60% on HS; The most common ESCr-E genera were *Enterobacter* (79%), *Pantoea* (10%), and *Klebsiella* (7%). ESCr-E were found in 2% of IF. HS most notably colonized with ESCr-E were the kitchen sink faucet (9%), oven door handle (4%), sofa (4%), bathroom sink faucet (3%) and bed sheets (3%), (Figures 1-3). ESBL-E were recovered from ∼2% of HHs. MDR-E (> 3 antibiotic classes) were found in 4% of HHs.

WGS of 20 *E. cloacae* from 4 HHs revealed within HH clusters (Figure 4). Of interest, we found that among *E. cloacae*, 10/20 (50%) contained AR plasmids (ex. IncFI, IncFII, IncX5, IncHI2, Col440I); *bla*, other AR (ex. PMQR, aminoglycoside, sulfa, fosfomycin) and virulence genes were detected in *E. cloacae* in all HHs. We also found related *E. cloacae* strains in 2 HHs (ST108), indicating local strains circulating potentially related to a common exposure.

**Figure 3.**
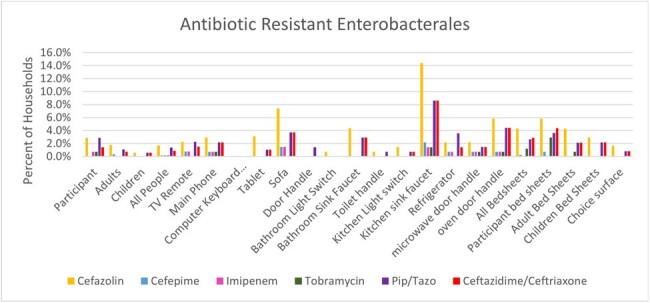
AR Enterobacterales by surface.

**Conclusion:**

ESCr-E are present in healthy household environments. Future directions will be to identify candidate clinical and epidemiological features linked with ESCr-E HH colonization.

**Figure 4.**
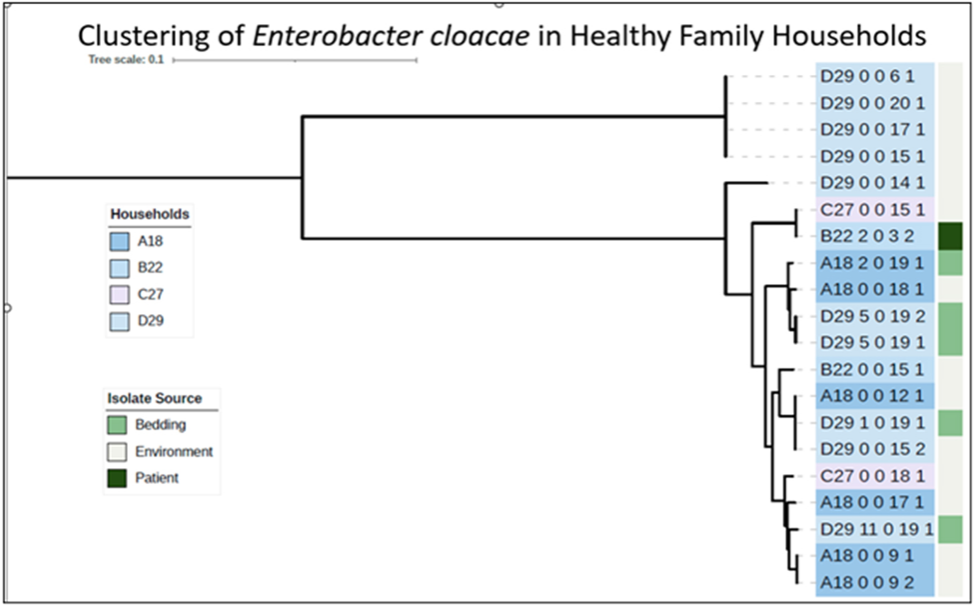
Genetic relatedness among household Enterobacter cloacae

**Disclosures:**

**Ahmed Babiker, MBBS**, Beckman Coulter Inc.: Advisor/Consultant

